# A Treatise on Diseases of the Eye

**Published:** 1891-07

**Authors:** 


A Treatise on Diseases of the Eye; for the Use of Students and General Practitioners. To which is added a series of test types for determining the exact state of vision. By Henry C. Angell, M. D., Professor of Ophthalmology in the Boston University School of Medicine. Seventh edition, re-written and illustrated. Boston: Otis Clapp & Son, 1891. Price, $3.00.

In Vol. II, page 286, HOMŒOPATHIC PHYSICIAN will be found what we thought of the sixth edition of the above "Treatise." This, the seventh edition, contains just what we condemned in the sixth—that is, allopathic teaching under the name of a professed homœopathist. We have had sufficient experience in various affections of the eyes to be able to state that Hahnemann's teachings apply to eye diseases as well as to other ailments, and that the results of applying these teachings are all that can be desired. In contrasting the results of a rigid adherence to homœopathic law in treating eye diseases with the haphazard methods of allopathy, we can only wonder at the short-sightedness of those who profess to know of Homœopathy but who will yet resort to the uncertainties of topical applications of crude drugs, to say nothing of their harm. While the book before us contains nothing new, still one can find in its pages some things worth knowing. The printer and binder are to be commended for their part of the work. G. H. C.

